# 524 Nutritional Risk in Frostbite

**DOI:** 10.1093/jbcr/irae036.159

**Published:** 2024-04-17

**Authors:** Caran Graves, Christopher R LaChapelle, Callie M Thompson, Irma D Fleming, Giavonni M Lewis

**Affiliations:** University of Utah Health Burn Center, Salt Lake City, UT; University of Utah, Salt Lake City, UT; University of Utah Health, Salt Lake City, UT; University Of Utah Burn Critical Care, Salt Lake City, UT; University of Utah Health Burn Center, Salt Lake City, UT; University of Utah, Salt Lake City, UT; University of Utah Health, Salt Lake City, UT; University Of Utah Burn Critical Care, Salt Lake City, UT; University of Utah Health Burn Center, Salt Lake City, UT; University of Utah, Salt Lake City, UT; University of Utah Health, Salt Lake City, UT; University Of Utah Burn Critical Care, Salt Lake City, UT; University of Utah Health Burn Center, Salt Lake City, UT; University of Utah, Salt Lake City, UT; University of Utah Health, Salt Lake City, UT; University Of Utah Burn Critical Care, Salt Lake City, UT; University of Utah Health Burn Center, Salt Lake City, UT; University of Utah, Salt Lake City, UT; University of Utah Health, Salt Lake City, UT; University Of Utah Burn Critical Care, Salt Lake City, UT

## Abstract

**Introduction:**

People with frostbite are often unhoused and have a history of substance use which increases risk of malnutrition. In this retrospective study, we sought to evaluate malnutrition risk and incidence in those with frostbite admitted to an academic medical center.

**Methods:**

Medical records of adults admitted with frostbite (ICD codes T33, T34) in 2019-2021 and with stays >4 days were reviewed. Data included anthropometrics, housing status, substance use and nutritional status. Patients were screened for nutritional risk using a modified malnutrition screening tool which focused on recent weight loss. High risk patients received an in-depth nutritional assessment. Nutritional interventions provided were also noted.

**Results:**

A total of 109 patients were admitted with frostbite and 42 (93% male) met inclusion criteria. Most had stable housing (69% housed; 31% unhoused) and 90% had insurance (mainly Medicare/Medicaid). Patient-reported alcohol (33%) and drug use (29%) were common among both housed and unhoused. Housed patients reported higher use of alcohol than unhoused (43% vs 6%) with similar reported use of other drugs. Three patients were at high risk for malnutrition based on recent weight loss. The remainder did not meet high risk screening criteria as 31 denied weight loss and 8 did not know. Five patients (12%) received a focused malnutrition evaluation but only three (all with reported weight loss) met criteria for malnutrition. All patients tolerated oral diets and 6 (14%) received supplemental enteral nutrition; no one required parenteral nutrition.

**Conclusions:**

This study population included a higher percentage of unhoused people than our usual thermal burn injured population. Frostbite patients also commonly used alcohol and drugs. Housing status and substance use are frequently cited risks for malnutrition, however, we did not identify malnutrition often in this population. Weight loss is a major risk factor for malnutrition and given the paucity of reported pre-admit weight loss this risk factor may have limited use for referral for a more focused nutritional evaluation.

**Applicability of Research to Practice:**

Few patients had malnutrition identified on admission which is inconsistent with published reports. Relying on self-reported weight and intake may obscure risk in this population and presents opportunity for more focused screening in our patients.

